# Patient Blood Management Program Implementation: Comprehensive Recommendations and Practical Strategies

**DOI:** 10.21470/1678-9741-2024-0205

**Published:** 2024-07-31

**Authors:** Isabel Cristina Céspedes, Maria Stella Figueiredo, Nelson Americo Hossne Junior, Ítalo Capraro Suriano, Rita de Cássia Rodrigues, Melca Maria Oliveira Barros, Manoel Antonio de Paiva Neto, Fernanda Chohfi Atallah, Bárbara Burza Benini, Adriano Miziara Gonzalez, Fábio Veiga de Castro Sparapani, Newton de Barros Júnior, Ieda Aparecida Carneiro, Celina Mayumi Morita Sarto, Caio Sussumu de Macedo Motoyama, Leonardo Sacchi, Victor Piovezan, Simone Luna de Almeida, Laís da Silva Pereira-Rufino, Solange Guizilini, Isadora Salvador Rocco, Nacime Salomão Mansur, Jaquelina Sonoe Ota Arakaki, Antonio Alceu dos Santos, Carlos Eduardo Panfilio

**Affiliations:** 1 Department of Morphology and Genetics, Discipline of Genetics, Escola Paulista de Medicina, Universidade Federal de São Paulo (UNIFESP), São Paulo, São Paulo, Brazil; 2 Department of Clinical and Experimental Oncology, Discipline of Hematology and Hemotherapy, Escola Paulista de Medicina, Universidade Federal de São Paulo (UNIFESP), São Paulo, São Paulo, Brazil; 3 Department of Surgery, Discipline of Cardiovascular Surgery, Escola Paulista de Medicina, Universidade Federal de São Paulo (UNIFESP), São Paulo, São Paulo, Brazil; 4 Department of Neurology and Neurosurgery, Discipline of Neurosurgery, Escola Paulista de Medicina, Universidade Federal de São Paulo (UNIFESP), São Paulo, São Paulo, Brazil; 5 Department of Anesthesiology, Pain and Intensive Care, Discipline of Anesthesiology, Escola Paulista de Medicina, Universidade Federal de São Paulo (UNIFESP), São Paulo, São Paulo, Brazil; 6 Department of Anesthesiology, Pain and Intensive Care, Discipline of Intensive Care, Escola Paulista de Medicina, Universidade Federal de São Paulo (UNIFESP), São Paulo, São Paulo, Brazil; 7 Department of Surgery, Discipline of Surgical Gastroenterology, Escola Paulista de Medicina, Universidade Federal de São Paulo (UNIFESP), São Paulo, São Paulo, Brazil; 8 Hospital São Paulo, Hospital Universitário da Universidade Federal de São Paulo (UNIFESP), São Paulo, São Paulo, Brazil; 9 Departments of Inventory and Pharmacy, Associação Paulista para o Desenvolvimento da Medicina (SPDM), São Paulo, São Paulo, Brazil; 10 Department of Medicine, Discipline of Pulmonology, Escola Paulista de Medicina, Universidade Federal de São Paulo (UNIFESP), São Paulo, São Paulo, Brazil; 11 Department of Clinical and Experimental Oncology, Postgraduate Program in Medicine - Hematology and Oncology, Escola Paulista de Medicina, Universidade Federal de São Paulo (UNIFESP), São Paulo, São Paulo, Brazil; 12 Escola da Saúde, Universidade Municipal de São Caetano do Sul (USCS), São Caetano do Sul, São Paulo, Brazil

**Keywords:** Blood Transfusion, Guideline, Health Strategies, Hospitalization, Costs and Cost Analysis, Research Personnel, Tertiary Care Centers, Public Health

## Abstract

**Introduction:**

Blood transfusion is one of the most common medical practices worldwide. However, current scientific literature has shown that the immunomodulatory effects of blood transfusion are associated with an increased likelihood of infection, prolonged hospitalization, and morbimortality. Also, it means high costs for healthcare systems.

**Methods:**

In this context, acknowledging that blood transfusions are essentially heterologous cell transplantations, the use of therapeutic options has gained strength and is collectively known as the patient blood management (PBM) program. PBM is an approach based on three main pillars: (1) treating anemias and coagulopathies in an optimized manner, especially in the preoperative period; (2) optimizing perioperative hemostasis and the use of blood recovery systems to avoid the loss of the patient's blood; (3) anemia tolerance, with improved oxygen delivery and reduced oxygen demand, particularly in the postoperative period.

**Results:**

Current scientific evidence supports the effectiveness of PBM by reducing the need for blood transfusions, decreasing associated complications, and promoting more efficient and safer blood management. Thus, PBM not only improves clinical outcomes for patients but also contributes to the economic sustainability of healthcare systems.

**Conclusion:**

The aim of this review was to summarize PBM strategies in a comprehensive, evidence-based approach through a systematic and structured model for PBM implementation in tertiary hospitals. The recommendations proposed herein are from researchers and experts of a high-complexity university hospital in the network of the Sistema Único de Saúde, presenting itself as a strategy that can be followed as a guideline for PBM implementation in other settings.

## INTRODUCTION

**Table:** 

Abbreviations, Acronyms & Symbols				
ACT	= Activated clotting time		ISS	= Injury Severity Score
CPB	= Cardiopulmonary bypass		IV	= Intravenous
EPO	= Erythropoietin		LDF	= Leukocyte depletion filter
EPM	= Escola Paulista de Medicina		PBM	= Patient blood management
Hb	= Hemoglobin		PTT	= Partial thromboplastin time
HU	= Hospital São Paulo		SC	= Subcutaneous
ICU	= Intensive care unit		SUS	= Sistema Único de Saúde
INR	= International Normalized Ratio		UNIFESP	= Universidade Federal de São Paulo

Historically, allogeneic blood transfusion has become one of the most common medical practices worldwide, largely due to its development and widespread use during the World Wars^[1]^. Unfortunately, it has been prescribed without the proper scrutiny by physicians, supposedly triggered by the low prevalence and severity of transfusion reactions, especially those of greater severity, suggesting it is a safe therapeutic resource. However, the advancement of molecular scientific methods has identified increasingly deleterious effects of blood transfusions on the recipients, leading to increased morbidity and mortality. The immunomodulation related to allogeneic blood transfusion (transfusion-related immunomodulation) brings significant consequences for the patient through mechanisms not yet fully understood. These mechanisms have been associated with the diminished function of natural killer cells and antigen-presenting cells, a reduction in cell-mediated immunity, and an increase in regulatory T cells^[2]^. Through these immunomodulatory and inflammatory effects, allogeneic blood transfusions have been associated with a higher risk of infection, prolonged hospital stays, and increased morbidity and mortality, regardless of the presence of confounding factors, such as demographic characteristics, healthcare team, hospital infrastructure, and patient conditions^[1,3]^.

Furthermore, with an aging population, a significant reduction in blood bank stocks is anticipated, which will force medicine to operate without allogeneic blood transfusions in many scenarios^[4]^. Additionally, allogeneic blood transfusions represent high costs for healthcare systems when we account for their production, transport, storage, and hospital administration. This cost burden is borne not only by the public healthcare system (Sistema Único de Saúde [SUS] in Brazil) per unit of blood but also by private healthcare entities. The prolonged hospital stays and morbidities associated with allogeneic blood transfusions further increase these costs^[5]^. In 2023, the Joint Commission reported that in American hospitals, 86.48% of transfusions were inappropriate or unnecessary, costing millions of dollars^[6]^.

In this context, strategies aimed at reducing or eliminating the use of allogeneic blood transfusions must be implemented. They are collectively known as patient blood management (PBM), with recommendations applied to both clinical and surgical patients. For surgical patients, these strategies are grouped into three pillars according to the perioperative period: preoperative, intraoperative, and postoperative. Simplified schemes of these strategies can be found in Farmer et al.^[4]^ or Santos et al.^[7]^.

A successful example of PBM implementation was observed in Australia, with a reduction in the use of blood components (41% in red blood cell concentrates, 47% in plasma, and 27% in platelet concentrates), resulting in 28% reduction in mortality, 31% reduction in acute myocardial infarction/stroke, 15% reduction in length of hospital stay, and 21% reduction in the infection rate, along with approximately AU$100 million savings in direct and indirect costs^[8]^. After 20 years of successful PBM implementation in Canada, significant reductions in hospital stay and infection rates were observed in cardiac and orthopedic surgery, saving around 50 million Canadian dollars per year^[9]^.

In 2021, the World Health Organization (WHO) declared that the implementation of PBM in hospitals worldwide is urgently needed^[8]^.

Therefore, the aim of the present review is to summarize PBM strategies using a comprehensive, evidence-based approach through a systematic and schematic model for PBM implementation in tertiary hospitals.

## PBM Pillars

PBM is a program that encompasses various models and strategies, depending on the authors or guidelines used, generally presented to constitute three pillars^[4,7]^:

- *1st pillar* (focus on the preoperative period) - optimizing erythrocyte mass and coagulation: primarily involves treatment of anemias and coagulopathies and patient preparation for surgical procedures, including nutritional aspects.

- *2nd pillar* (focus on the intraoperative period) - minimizing blood loss: involves preserving the patient's blood, minimizing its loss during surgery, and optimizing coagulation and surgery hemostasis through the use of systemic and topical hemostatic, surgical hemostatic instruments, blood cell recovery, and/or acute normovolemic hemodilution.

- *3rd pillar* (focus on the postoperative period) - tolerance to anemia: involves the concept of anemia tolerance through measures aimed at enhancing tissue oxygen delivery, sedation, and analgesia optimization to reduce oxygen consumption, minimizing phlebotomy frequency and volume, maintaining normothermia, and optimizing cardiac output through volume expanders when appropriate.

In Brazil, most diagnostic strategies, pharmacological treatments (for anemias, coagulopathies, hemostasis, equipment kits), and necessary equipment (blood cell recovery, viscoelastic testing) for PBM implementation are included in the Relação Nacional de Medicamentos (RENAME or National List of Medicines) and Relação de Equipamentos e Materiais (RENEM or List of Equipment and Materials) of the SUS, which ensures feasibility of implementation in hospitals^[7]^.

It is important to point out that PBM represents a paradigm shift that recognizes the transfusion of blood components as a transplantation of heterologous tissue, with all its implications and complications, and should not be confused with restrictive blood strategy regimens or merely the treatment of anemias.

## METHODS

### Proposal for PBM Implementation

The recommendation to implement PBM emerged from an initiative by managers, specialists, and researchers of the Escola Paulista de Medicina (EPM)/Universidade Federal de São Paulo (UNIFESP)/Hospital São Paulo (HU/UNIFESP). As these professionals delved deeper into the subject, they recognized its relevance, particularly highlighting the increased patient safety, efficient use of public health financial resources, and concern over the postponement of surgical procedures due to shortages of blood components in blood banks. Furthermore, they acknowledged the aging population in Brazil, which is expected to further reduce the availability of donors, along with potential new pandemics of bloodborne transmission that may arise.

### Step-by-Step Implementation Recommendation: 10-Step Plan

#### 1st step - Initiative: Personal Motivators

Many medical and non-medical managers, hematologists, clinicians, surgeons, obstetricians, anesthetists, intensivists, nurses, or biomedical professionals may have been exposed to the PBM and the compelling arguments supporting its urgent implementation. This first step is to approach the hospital management team or strategic partners, such as transfusion agencies and/or heads of surgical, intensive care, or anesthesia divisions, so as to introduce the subject and identify potential pathways for PBM implementation within their hospital context.

#### 2nd step - To Create a Working Group

The implementation of a PBM program, regardless of its description depicted here as a staged strategy, involves the entire patient care continuum and, hence, multiple sectors within the hospital environment. Moreover, it entails several aspects, like defining areas where it should be implemented, developing well-established protocols and processes, monitoring and auditing the implementation progress - and adjusting as necessary -, and requires the consolidation of a representative Working Group of several hospital divisions. This Working Group can become a permanent and ongoing advisory group.

#### 3rd step - To Define Pilot Areas

Following its formation, the Working Group must identify one or two pilot surgical areas for PBM implementation. This approach allows for focused efforts on those areas that can serve as internal and accreditation models. The selection should be based on technical considerations and the willingness of specific divisions to undertake the initial implementation efforts and paradigm shifts.

#### 4th step - Local Diagnosis: Knowledge, Infrastructure, and Personnel

Upon forming the Working Group and selecting pilot areas, clear and objective actions must be planned to engage key stakeholders and environments involved in PBM implementation, focusing on physicians, nurses, and administrators. Therefore, a local diagnostic assessment is necessary, which can be conducted through clear and objective questions in an electronic form.

This questionnaire should include inquiries about:

a) how critically teams analyze the risks and benefits of allogeneic blood transfusions before prescribing and administering them, their knowledge of PBM, and their readiness to change their practice; and

b) the local infrastructure available in terms of diagnostic tests, medications (for anemias, coagulopathies, bleeding), equipment (blood cell recovery, viscoelastic testing), and human resources that the hospital possesses.

#### 5th step - To Sensibilize and to Capacitate

Based on the level of knowledge revealed through the assessment of item “a” of the 4th step, to promote: i) one or more meetings with teams from pilot areas to align them with PBM implementation; ii) training courses on practical actions in PBM implementation. This step is of paramount importance. Had there been training in PBM since undergraduate studies or an institutionalized training program, PBM would already be naturally integrated into medical and hospital routines, significantly reducing many of the current implementation efforts. Education, indeed, is the primary driving force for PBM to take effect.

#### 6th step - To Define PBM Strategies and to Create Protocols

Each hospital setting has its characteristics, strengths, and challenges in implementing PBM, which can be identified through the assessment of item “b” of the 4th step. Therefore, based on possible PBM strategies as suggested by Farmer et al.^[4]^ and Santos et al.^[7]^, the Working Group must discuss and define which PBM strategies to incorporate locally. Consideration should be given not only to available infrastructure and human resources but also to how much hospital management is willing to invest in these dimensions, considering that the PBM program will significantly lower costs through reduced blood component usage and fewer clinical complications, among others. Care protocols must be developed for the strategies of the 1st pillar (management of anemias and coagulopathies), 2nd pillar (use of topical and systemic hemostatic, blood cell recovery, acute normovolemic hemodilution, viscoelastic testing), and 3^rd^ pillar (tolerance and management of anemia and postoperative bleeding with the use of viscoelastic testing to identify causes). This is also a crucial step for effective real-world implementation of PBM. These care protocols provide immediate, accurate, and practical decision-making derived from evidence-based studies.

#### 7th step - To Define a Start Date

Ensure all previous steps are consolidated and define a start month for implementation to collect retrospective (pre-PBM) and prospective (post-PBM) data.

#### 8th step - Fortnightly Meetings

Close monitoring of PBM implementation actions is necessary through SWOT (or strengths, weaknesses, opportunities, threats) analyses and continuous adjustments and adaptations.

#### 9th step - Outcomes Evaluation

Through the hospital’s electronic medical records or database, extract anonymous data on key outcomes to be analyzed post-PBM implementation, such as quantity and type of perioperative blood components used, infection rates, postoperative length of stay, reoperations, inflammatory markers, postoperative complication, and mortality in the implemented area.

#### 10th step - Compliance and Results Auditing

In addition to technical and financial evaluations directly related to implementation controlled by the Working Group, ensuring the effectiveness and compliance of PBM implementation recommends regular and systematic audits through an Advisory Committee formed within the Working Group (at least two of its members), including a representative from the hospital’s patient safety or quality department and an administrative director representative. These audits should include:

- Definition of performance indicators: to establish clear and measurable indicators, such as reduced use of blood components, decreased postoperative complications, shorter hospital stays, reduced infection rates, reduced mortality, and cost-effectiveness analysis. This work can be done collaboratively with the Working Group.

- Ongoing monitoring: to implement a continuous monitoring system to track performance indicators, allowing for timely identification of deviations and ensuing corrective actions.

- Compliance assessment to conduct periodic audits: to verify adherence to PBM guidelines, including reviewing medical records, analyzing clinical protocols, and directly observing transfusion practices.

- Feedback and continuous improvement: to establish a feedback mechanism involving healthcare and administrative teams, promoting continuous improvement based on audit results and identified best practices.

- Performance reporting: to develop regular reports summarizing audit and monitoring results, highlighting areas of success and opportunities for improvement.

By implementing these audit and compliance steps, it will be possible to ensure that PBM is effectively applied following established guidelines, promoting patient safety and efficiency in healthcare resource utilization.

Note that the recommended steps for PBM implementation may change, as some steps may occur concurrently or in multiple stages depending on the dynamics or challenges of each site.

## RESULTS

### Strategic Actions in the PBM Implementation - Efficient Practices

Based on the step-by-step recommendation outlined previously, the Working Group known as PBM-HU-UNIFESP (https://pbm.unifesp.br/), responsible for PBM implementation at HU/UNIFESP, following extensive analysis of current scientific literature and intensive discussions among involved areas and experts, recommends the application of the following practices:

Regarding the *1st step* and the initial role of individual motivators in overcoming challenges in PBM implementation, it is essential that motivated and dedicated leadership with expertise in PBM must activate the hospital clinical board, which should take an active role by facilitating initial discussions and serving as a link between potential areas and their representatives. In this case, for example, EPM/UNIFESP had the initiative of a faculty leader.

The practical recommendation for the *2nd step* is to establish a Working Group, considering the crucial need for multidisciplinary collaboration in enabling PBM and ensuring all perspectives and concerns are addressed. Like the PBM-HU-UNIFESP Group example, this Working Group should include members from the Transfusion Committee and heads/representatives from Hematology and Blood Therapy focusing on the 1st pillar; heads/representatives initially from one or two surgical areas and from Anesthesiology focusing on the 2nd pillar; and coordinators/representatives from intensive care units (ICUs) focusing on the 3rd pillar. Important for all pillars, the Working Group should include nursing management, supplies, and devices department (due to demands for diagnostic tests and medications for anemia, coagulopathies, hemostasis, and disposable kits for equipment), clinical engineering (to ensure the feasibility and maintenance of equipment such as blood cell recovery and viscoelastic testing), and a representative from medical residency. This group has the potential to expand as PBM extends to areas beyond those initially involved, and ideally, it should be permanent and integrated with the hospital’s clinical board.

For the *3rd step*, it is recommended, as in the practical example of the PBM-HU-UNIFESP Group, that two surgical areas be initially selected as pilot implementation areas (in our implementation, cardiovascular surgery and neurosurgery). Two pilot areas allow for the identification of the root cause of possible difficulties, whether they are related to the PBM or specifically to that surgical division. Screening and selection of these pilot areas should include those with a high risk of perioperative blood loss and those demonstrating immediate interest in facilitating this initial phase. Experience gained in these pilot areas allows for a structured advancement to other areas, ensuring a gradual and successful PBM implementation throughout the entire hospital.

For the *4th step*, it is recommended that the Working Group thoroughly understand the hospital’s scenario regarding PBM and its technical and administrative aspects. As in the PBM-HU-UNIFESP Group example, comprehensive assessment tools should be used, such as scientifically based and user-friendly questionnaires (multiple-choice questions with space for detailed responses). These questionnaires should be electronically administered to healthcare teams in pilot areas and include questions about: a) knowledge regarding transfusion practice and PBM based on its principles; and b) available infrastructure and personnel, as per hospital accreditation manual guidelines (Manual Brasileiro de Acreditação Hospitalar; Brazil)^[10]^. Analysis of obtained information will identify training needs and pitfalls, especially regarding allogeneic blood transfusion uses and PBM practices. Studies indicate insufficient training in transfusion medicine and PBM in medical schools in Brazil and abroad^[11,12]^. Following the PBM-HU-UNIFESP Group’s experience, this evaluation instrument can be made available through agreements or partnerships with interested institutions, facilitating the identification of specific hospital needs and enabling more precise and effective future planning. Performing such tools is fundamental to tailoring PBM to each institution’s specificities, promoting better clinical and operational outcomes.

The practical recommendation for the *5th step* is to diagnose the team's knowledge on the subject (item “a” from the 4th step). From then on, it is imperative to effectively communicate the importance of PBM, since not all healthcare professionals directly involved in patient care are familiar with or actively engaged it. Therefore, developing communication strategies that clarify the clinical, surgical, and operational benefits of PBM using scientific data and practical examples to engage the entire team is essential. Promoting workshops, seminars, and interactive discussions can help increase awareness and engagement, enhancing program adherence and ensuring its success, particularly in involved pilot areas. The PBM-HU-UNIFESP Group already has a training system that began with the inclusion of PBM in the undergraduate Medicine course at EPM as an elective discipline, and a mandatory PBM training course for all medical residents at HU/UNIFESP through the Comissão de Residência Médica (COREME or Medical Residency Committee at EPM). It is recommended that training courses include specialists addressing PBM applied to various areas such as hematology, anesthesiology, surgical specialties, obstetrics, pediatrics, trauma, and interprofessional practice. Through partnerships or agreements, this course can be offered to other interested entities/hospitals.

**Fig. 1:**
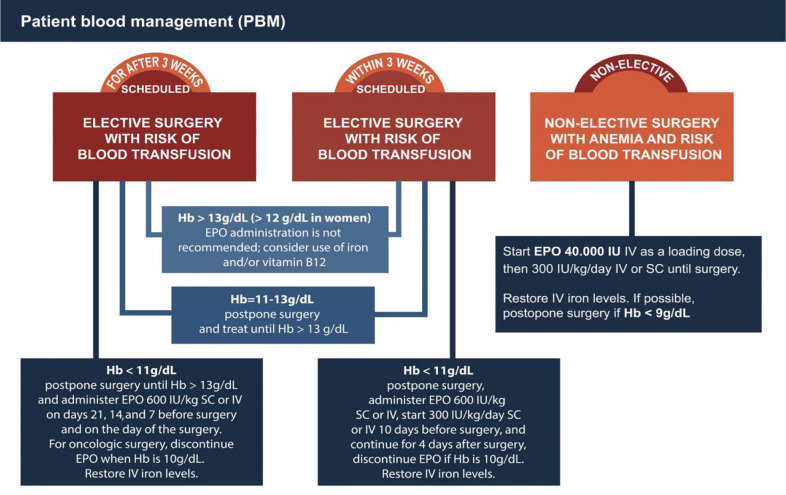
Simplified scheme of the Pharmacological Treatment of Anemia Protocol and Guidelines for Erythropoietin Therapy. EPO=erythropoietin; Hb=hemoglobin; IV=intravenous; SC=subcutaneous.

**Fig. 2:**
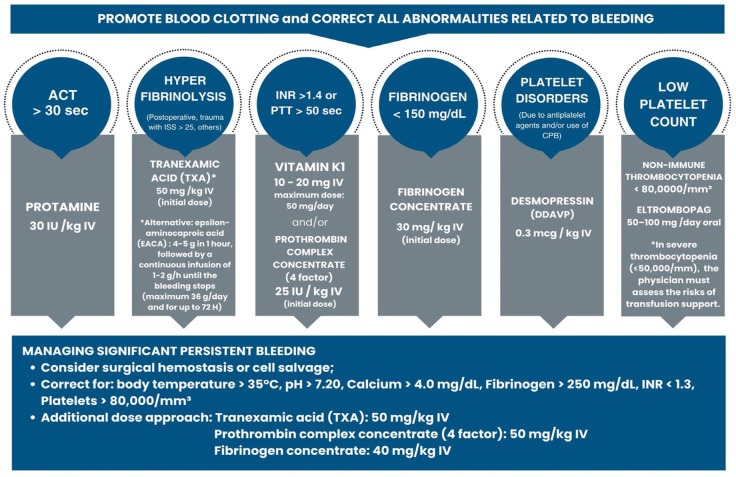
Simplified scheme of the Bleeding Management Protocol. Algorithm for persistent bleeding. ACT=activated clothing time; CPB=cardiopulmonary bypass; INR=International Normalized Ratio; ISS=Injury Severity Score; IV=intravenous; PTT=partial thromboplastin time.

For the *6th step*, the practical recommendation is that actions can be planned based on the infrastructural and personnel conditions identified by the local assessment (item “b” of the 4th step). Like the PBM-HU-UNIFESP Group example, based on assessment results, local resources available for diagnosis and treatment of anemias and coagulopathies, systemic and topical hemostatic, blood cell recovery, and viscoelastic testing should be listed. Furthermore, it is important to verify the availability of medical, nursing, and technical personnel, residents, and administrative staff who can incorporate PBM into their routines and support or engage in its implementation. Initially, specific hiring for PBM is not anticipated, considering its implementation involves practice and mindset changes. Reorganizing routines and personnel have proven feasible in the PBM-HU-UNIFESP Group example. Through local diagnosis, an important step is developing evidence-based care protocols that include PBM strategies applicable locally and ensuring implementation effectiveness. As an example from the PBM-HU-UNIFESP Group, actions are highlighted in the following items:

*a) Development of Protocols:* care protocol recommendations were developed after extensive analysis of current scientific literature and intense discussions among the Working Group members and involved areas, including the Pharmacological Treatment of Anemia Protocol and Guidelines for Erythropoietin Therapy (Figure 1) and the Bleeding Management Protocol (Figure 2). These are clear and objective care protocol recommendations for clinical and surgical practice, readily available for those institutions interested in implementation.

*b) Preoperative Care:* Hematology and Blood Therapy Division and the Blood Center emphasized the relevance of creating a PBM anemia ambulatory. This ambulatory can operate by reorganizing existing services in these sectors, initially for one day a week, as done in the example of PBM implementation at HU/UNIFESP. This allows outpatients from pilot areas to be prepared for elective surgical procedures by treating preoperative anemias and coagulopathies based on PBM principles through the abovementioned Pharmacological Treatment of Anemia Protocol and Guidelines for Erythropoietin Therapy (section: elective surgical patients). The same team of professionals also assesses hospitalized patients in case of urgency and emergency surgeries through consultations, also following the Pharmacological Treatment of Anemia Protocol and Guidelines for Erythropoietin Therapy recommendations (section: non-elective surgical patients), with treatments optimized due to time constraints.

*c) Efficient Practices in Intraoperative Care:* establishment of intraoperative management practices involving Anesthesiology and Surgical areas, based on PBM principles through the aforementioned Bleeding Management Protocol, through collaboration between anesthesiology and surgical teams in order to optimize systemic and topical hemostatic use, blood cell recovery, and viscoelastic testing.

*d) Efficient Practices in Postoperative Care:* reinforcement of the concept of anemia tolerance among ICU intensivists responsible for postoperative care, based on individual patient’s condition. Important aspects include early nutrition (oral/enteral/parenteral) to support erythropoiesis, avoid iatrogenic blood losses (excessive blood collection for laboratory tests), and manage anemia and bleeding. In this case, viscoelastic testing is also relevant for identifying causes of potential bleeding.

An initial investment is required for acquiring new drugs for anemia treatment and hemostatic, as well as acquiring blood cell recovery and viscoelastic testing devices outside the hospital’s normal routines. However, it should be highlighted that by reducing the use of blood components, the costs will be significantly reduced as well.

The recommendations for the *7th step*, according to the PBM-HU-UNIFESP Group example, are to set a date for PBM implementation, to conduct an official inaugural event to reinforce the importance of team roles and supporting personnel, to organize necessary actions globally, and to establish a milestone for sharing information on implementation effectiveness.

The recommendation for the *8th step* is to maintain the Working Group’s actions, following the PBM-HU-UNIFESP Group example. In the initial phase, continuous monitoring of actions will be necessary, identifying difficulties, threats, or resistances, considering opportunities and strengths to overcome them, and allowing the process to progress effectively. Fortnightly meetings may suffice. This PBM management activity should also analyze the addition and proper integration timing of new hospital areas and divisions into the implementation process. In the case of PBM-HU-UNIFESP Group, managers have incorporated PBM as an institutional project for improving patient care pathways in various hospital processes, aiming to reduce morbidity, mortality, and costs.

The recommendation for the *9th step* is the need to systematize the acquisition of implementation-related data for technical evaluation, managed by the Working Group. To ensure the success of PBM implementation, it is essential to utilize computerized systems and establish systematic data collection from patients undergoing the protocol. These systems facilitate result analysis and monitoring of PBM impact over time. Annual evaluation of outcomes is the key, by defining performance indicators, statistical analyses, and engaging all stakeholders. This process identifies areas for continuous improvement and promotes patient care excellence through PBM. Moreover, implementing a monitoring and evaluation system measures adherence to PBM protocols and their impacts, allowing for timely adjustments and continuous improvements. These measures foster a collaborative, structured environment that enhances PBM adherence and improves clinical outcomes. Data acquisition should focus on metrics directly impacted by PBM implementation, as evidenced in the literature^[3,4,8]^. Patient safety-related metrics include: the use of blood components (type and number of units), infection rates, length of hospital stay, reoperations, vascular accidents, postoperative complications, and mortality, as recommended by PBM-HU-UNIFESP Group. These metrics can quantify resource savings through reduced blood component usage, shorter hospital stays, and fewer complications.

The recommendation for the *10th step* is for audit and compliance actions of the implementation to be conducted by an advisory committee linked to the clinical board, given their institutional control nature. It is recommended that this committee include at least two medical members from the Working Group, a representative from the quality assurance or patient safety divisions, an administrative management representative, and a nursing representative. This committee plays a bureaucratic role in organizing information, analyzing performance, balancing results, and supporting hospital management.

**Fig. 3:**
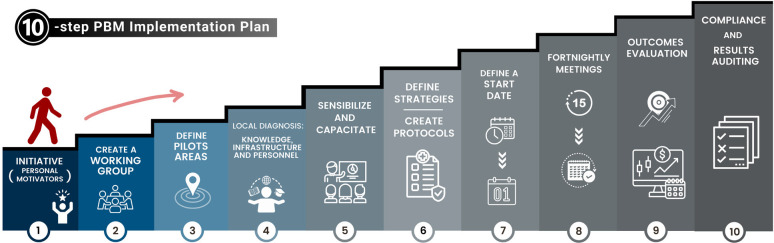
This diagram outlines the step-by-step implementation plan for the patient blood management (PBM).

All steps of PBM implementation are illustrated in Figure 3.

## DISCUSSION

### Recommendation Based on Evidence

The successful implementation of the PBM program depends heavily on the intense mobilization of those involved, particularly within the Working Group, which must motivate other stakeholders from pilot areas, as well as managers, residents, and administrative staff. This is because, as exemplified by the PBM-HU-UNIFESP Group, the implementation process began in an idealistic manner, driven by everyone’s commitment to improve patient safety and allocate public resources efficiently. In this example, all actions were carried out without any conflicts of interest, neither with product or equipment manufacturers or institutions supplying blood products. The PBM implementation process has led to a reassessment of patient care pathways, resulting in improvements in preoperative preparation, optimized surgical practices, and stricter criteria for blood component uses. In a university hospital setting, there has been notable scientific involvement from medical and nursing specialties, encouraging participation in existing institutional postgraduate research programs and further motivating residents. These actions have also facilitated the creation of a space for integration across different areas, fostering an environment for knowledge exchange and best practices. Thus, based on these recommendations that include an implementation experience report (PBM-HU-UNIFESP Group), the importance of motivation among all stakeholders becomes evident. As a result of that, it is strategic to engage not only managers but the entire healthcare team. In this regard, beyond the development of flows and processes, direct engagement with frontline healthcare professionals is the key to success. Above all, education stands out as the primary factor, as knowledge changes behavior.

Indeed, a recent study^[13]^ sought to analyze the motivations behind red blood cell concentrate prescriptions by anesthesiologists and surgeons, as these motivations drive decision-making and can explain the significant variability in practice among physicians and hospitals. Various aspects were identified, including: (1) knowledge, (2) social/professional role and identity, (3) beliefs about consequences, (4) environmental context/resources, (5) social influences, (6) behavioral regulation, (7) nature of the behaviors, and (8) memory, attention, and decision processes. It can be observed how educational and motivational efforts aimed at those involved in PBM implementation are more important than mere administrative decisions, given the myriad factors influencing these clinical decisions. Therefore, educational aspects should be considered as relevant as the need for administrative flows and processes.

The recommendation of the protocol cited and used by the PBM-HU-UNIFESP Group for Pharmacological Treatment of Anemia and Guidelines for Erythropoietin Therapy contains strategies for various scenarios, considering the main causes of anemia, surgical (elective and non-elective) and non-surgical patients, optimized forms of iron replacement, erythropoietin (EPO) use, and necessary precautions in the use of these medications. Indeed, the treatment of anemias plays a central role in PBM implementation. Warner et al.^[14]^ study in cardiac surgery demonstrated the association between preoperative anemia and worse outcomes, including acute kidney injury and increased length of hospital stay. Perioperative organ injuries caused by ischemic tissue anemia induce secondary inflammation and elevations in pro-inflammatory cytokines, which can be exacerbated by the inflammatory insult of extracorporeal circulation. Besides, preoperative anemia is a significant risk factor for perioperative red blood cell transfusion, which in turn is associated with adverse clinical outcomes. In this context, a recent update from the Society for the Advancement of Patient Blood Management^[15]^ emphasized the significant role of EPO (in conjunction with iron) in treating anemias for elective patients, becoming a valuable resource when there is limited time for non-elective surgery preparation. The report indicates that potential cardiovascular event risks associated with EPO do not occur, and it may even have a nephroprotective effect. Additionally, in cardiac surgery, the following events occur: beta-blockers suppress endogenous EPO production, and perioperative anemia diminishes the cardioprotective effect of beta-blockade; cytokines stimulated by inflammatory response associated with extracorporeal circulation limit EPO production; and perioperative renal ischemia may limit EPO production. Therefore, the use of EPO a few days before cardiac surgery becomes relevant for reducing red blood cell volume. Also, EPO use can significantly reduce the likelihood of perioperative red blood cell concentrate use (from 93% to 67%), and a single dose of 80,000 IU of EPO two days before surgery reduced postoperative red blood cell concentrate use by 22%. Consequently, studies show cost reduction in preoperative anemia treatment by reducing red blood cell concentrate use and associated perioperative complications^[1,8,9]^.

The recommendation of the abovementioned Bleeding Management Protocol used by the PBM-HU-UNIFESP Group contains the best guidelines and prescriptions for systemic hemostatic in various clinical-surgical scenarios, options and recommendations for topical hemostatic, the use of blood cell recovery devices, and acute normovolemic hemodilution. The use of synthetic antifibrinolytic agents, such as tranexamic acid or epsilon-aminocaproic acid, reduces blood loss and blood transfusion during cardiac procedures, trauma, heavy menstrual bleeding, peripartum hemorrhage, traumatic brain injury, and surgical site bleeding^[15]^. Indeed, its optimized use, within the logic of PBM as described in the protocol, is a powerful ally in bleeding control and avoids allogeneic blood transfusion use. Another important element for conserving the patient's own blood is the use of blood cell recovery. A review article by Ashworth & Klein^[16]^ analyzed the use of blood cell recovery, its benefits, risks, and possible complications in various scenarios such as cardiac and vascular surgery, neurosurgery, obstetrics, orthopedics, pediatrics, and oncology. As reported in the review, blood cell recovery is safe and effective in reducing blood transfusions in elective adult surgeries in the analyzed areas, particularly in cardiac and orthopedic surgeries. Its use should be considered in cases where significant blood loss (500 mL)^[17]^ is expected. Its use with a leukocyte depletion filter (LDF) appears safe in malignancy cases. In obstetric patients, the use of LDFs is not recommended. Additional studies are needed for consolidation in both cases. The only absolute contraindication to the use of blood cell recovery is patient refusal. This review also highlights the cost-effectiveness of blood cell recovery use compared to allogeneic blood transfusion. Its use has been shown to be cost-effective (with emphasis on cardiac and orthopedic surgeries), and data have already demonstrated savings of US$110.54 with the use of blood cell recovery compared to a unit of allogeneic blood transfusion^[16]^. In this context of cost-effectiveness, acute normovolemic hemodilution is a low-cost intraoperative technique for conserving the patient’s own blood. It is performed immediately before the surgical procedure and involves removing the patient’s total blood into empty blood bags, calculated individually while maintaining the patient's volume with crystalloid and/or colloid solutions. The removed blood is returned to the patient immediately after the surgical procedure. Acute normovolemic hemodilution has been successfully performed in cardiac surgeries since the 1970s. Currently, several studies have shown it to be safe, inexpensive, and effectively reducing the need for blood transfusions in abdominal surgeries^[18]^.

Regarding the change in the concept of anemia tolerance, a recent review poses an important question in its title: “RBC Transfusion Triggers: Is There Anything New?”^[19]^. This review highlights that for many years, the traditional 10/30 rule (hemoglobin 10 g/dL, hematocrit 30%) has been used as a trigger for allogeneic blood transfusions. It is now believed that this concept has contributed to countless unnecessary transfusions and an unknown number of deaths related to multiple transfusions. As pointed out by the authors, recent studies show that lower hemoglobin levels can be safely accepted, even in critically ill patients. Furthermore, even these new thresholds for restrictive transfusion are far beyond the theoretical limits of individual anemia tolerance. Although this concept may seem intuitive at first glance, the authors have demonstrated that there is no solid scientific evidence supporting the safety and benefit of relying solely on laboratory triggers to prescribe allogeneic blood transfusions. In this sense, this publication encourages us to continue seeking more sensitive and specific parameters regarding the overall clinical-surgical conditions of each patient, as studies involving patients who refuse blood transfusions show that the body indeed tolerates anemia more than physicians themselves.

Areas such as liver transplantation, now included in our implementation process, from the PBM-HSP-UNIFESP Group, have also benefited from PBM principles. A recent publication^[20]^ showed that allogeneic blood transfusions in liver transplantation significantly increase post-transplant morbidity and mortality and are associated with reduced graft survival. In this context, PBM emerges as an important alternative. In intensive care medicine, also included in the process of the example presented here, from the PBM-HSP-UNIFESP Group, patients present multiple risk factors for anemia and comorbidities, and PBM offers individualized strategies that significantly contribute to the management of anemia, coagulopathies, and iatrogenic blood loss^[21]^. In the pediatric population, a recent review points out the significant relevance of developing PBM programs for neonates and children and the challenges in this regard, demonstrating that the same concepts and parameters as those of PBM programs for adults should not be used. However, it is urgent that this area be included as soon as possible in the PBM strategy^[22]^.

As a final note, PBM brings better results for healthcare teams, patients, and vulnerable populations with micronutrient deficiencies, anemia, and/or bleeding. It also brings significant financial benefits to public and private hospitals, and healthcare systems. WHO has declared urgency in implementing PBM in its 194 member countries for patient safety, high costs of blood transfusions, aging populations, and potential new pandemics. According to Hofmann et al.^[5]^, the implementation of PBM involves the so-called 3Es - evidence, economy, and ethics - which fully justify the urgency of implementation.

### Limitations

As limitations, the economic outcomes of the implementation example presented here have not been included, which will be done in future publications.

## CONCLUSION

Current scientific evidence supports the effectiveness of PBM by reducing the need for blood transfusions, decreasing associated complications and mortality, and promoting more efficient and safer PBM. Thus, PBM not only improves clinical outcomes for patients but also contributes to the economic sustainability of healthcare systems. The implementation of this program may lead to optimized medical practices, yielding benefits for both patients and healthcare systems. Therefore, based on current evidence, the implementation and dissemination of PBM in hospitals and healthcare centers is strongly recommended. The aim of this review was to summarize PBM strategies in a comprehensive, evidence-based approach through a systematic and structured model for PBM implementation in tertiary hospitals. The recommendations proposed herein are from researchers and experts of a high-complexity university hospital in the SUS network, presenting itself as a strategy that can be followed as a guideline for PBM implementation in other settings.
